# *M. tuberculosis* Sliding β-Clamp Does Not Interact Directly with the NAD^+^ -Dependent DNA Ligase

**DOI:** 10.1371/journal.pone.0035702

**Published:** 2012-04-24

**Authors:** Vandna Kukshal, Taran Khanam, Deepti Chopra, Nidhi Singh, Sabyasachi Sanyal, Ravishankar Ramachandran

**Affiliations:** 1 Molecular and Structural Biology Division, Central Drug Research Institute (Council of Scientific and Industrial Research), Lucknow, Uttar Pradesh, India; 2 Drug Target Discovery and Development Division, Central Drug Research Institute (Council of Scientific and Industrial Research), Lucknow, Uttar Pradesh, India; University of Delhi, India

## Abstract

The sliding β-clamp, an important component of the DNA replication and repair machinery, is drawing increasing attention as a therapeutic target. We report the crystal structure of the *M. tuberculosis* β-clamp (Mtbβ-clamp) to 3.0 Å resolution. The protein crystallized in the space group C222_1_ with cell-dimensions *a* = 72.7, *b* = 234.9 & *c* = 125.1 Å respectively. Mtbβ-clamp is a dimer, and exhibits head-to-tail association similar to other bacterial clamps. Each monomer folds into three domains with similar structures respectively and associates with its dimeric partner through 6 salt-bridges and about 21 polar interactions. Affinity experiments involving a blunt DNA duplex, primed-DNA and nicked DNA respectively show that Mtbβ-clamp binds specifically to primed DNA about 1.8 times stronger compared to the other two substrates and with an apparent K_d_ of 300 nM. In bacteria like *E. coli*, the β-clamp is known to interact with subunits of the clamp loader, NAD^+^ -dependent DNA ligase (LigA) and other partners. We tested the interactions of the Mtbβ-clamp with MtbLigA and the γ-clamp loader subunit through radioactive gel shift assays, size exclusion chromatography, yeast-two hybrid experiments and also functionally. Intriguingly while Mtbβ-clamp interacts *in vitro* with the γ-clamp loader, it does not interact with MtbLigA unlike in bacteria like *E. coli* where it does. Modeling studies involving earlier peptide complexes reveal that the peptide-binding site is largely conserved despite lower sequence identity between bacterial clamps. Overall the results suggest that other as-yet-unidentified factors may mediate interactions between the clamp, LigA and DNA in mycobacteria.

## Introduction

The sliding β-clamp polymerase processivity factor adopts a doughnut-like ring shaped structure where the DNA passes through the positively charged inner hollow of the clamp [1]. It was earlier thought that the β-clamp does not exhibit specific interactions with DNA until recently when experiments involving the *E. coli* β-clamp demonstrated that these proteins exhibit specific binding to blunt dsDNA and ssDNA respectively [2]. The β-clamp itself has to be loaded onto DNA with the help of the multi-protein clamp loader complex [3]. β-Clamps are essential for many important DNA transactions including replication and repair. It recruits various factors like DNA polymerases, ligases, and other DNA interacting/processing proteins to the ‘scene of action’. The co-crystal structure of the *E. coli* β-clamp with DNA revealed that the DNA is tilted by about 22° compared to the plane of the clamp ring and this suggested a model where the DNA tilts from one subunit of the clamp to the other, presumably to interact with different factors bound to the clamp. Analogous electron microscopy and single particle image analysis studies involving the *P. furiosus* proliferating cell nuclear antigen (PCNA), ligase and DNA demonstrated that the DNA is tilted by about 16° when it passes through PCNA [4]. It therefore appears that tilting of DNA through the clamp/PCNA is a mechanism adopted by the protein to facilitate its interactions with the other factors.

Interactions of the *E. coli* clamp with proteins like the LigA and components of the clamp loader complex have been reported earlier [5]. This is similar to the strong interactions of the PCNA with DNA ligase I, FEN-1 and other proteins reported earlier [6]–[7]. Other reports involving crystal structures and small angle X-ray scattering experiments [8] have afforded insights as to how the ligase retains its mobility despite interacting with the PCNA. Analogous experiments involving bacterial clamps are yet to be reported.

Structures of β-clamps are known from diverse sources including *E. coli, T. maritima and S. pyogenes* till date [1], [9]. These structures show that the β-clamp exists as homodimers whose protomers assemble in a head-to-tail fashion. PCNA on the other hand is a trimer. Structural differences between the bacterial and human homologs and also the important nature of the β-clamp makes it a potential drug target. Indeed, one group has reported the co-crystal structure of an inhibitor with the *E. coli* β-clamp where the binding site of the inhibitor overlaps with the region to which DNA polymerases bind [10].

Our group has been working on the structural characterization of the *M. tuberculosis* LigA (MtbLigA) and the identification of inhibitors that are specific for it compared to the human DNA ligase I [11]–[13]. We were interested in analyzing the structural determinants of the interactions of MtbLigA with the Mtbβ-clamp expecting them to be analogous to the reports involving PCNA and DNA ligase I as also the *E. coli* LigA and its β-clamp. Surprisingly we found that no direct interactions exist *in vitro* between MtbLigA and Mtbβ-clamp. On the other hand control experiments confirm that the *E. coli* LigA and its clamp interact with each other. Presumably other unknown/as-yet-unidentified factors other than the target DNA should mediate the interactions between these proteins. Mtbβ-clamp itself exhibits affinity for components of the clamp loader complex. Structurally it is similar to those of the clamps from other sources like *E. coli, S. pyogenes* and *T. maritima*. The crystal structure reveals the details of the positively charged inner ring that is involved in DNA recognition and the hydrophobic binding site that is involved in the recognition of partner proteins. On the other hand the affinity experiments highlight differences in its affinity for primed DNA, blunt dsDNA and nicked DNA compared to earlier reports involving the *E. coli* β-clamp.

## Results

### Characterization of the Mtbβ-Clamp affinity for primed DNA, blunt dsDNA and nicked DNA respectively

**Figure 1:**
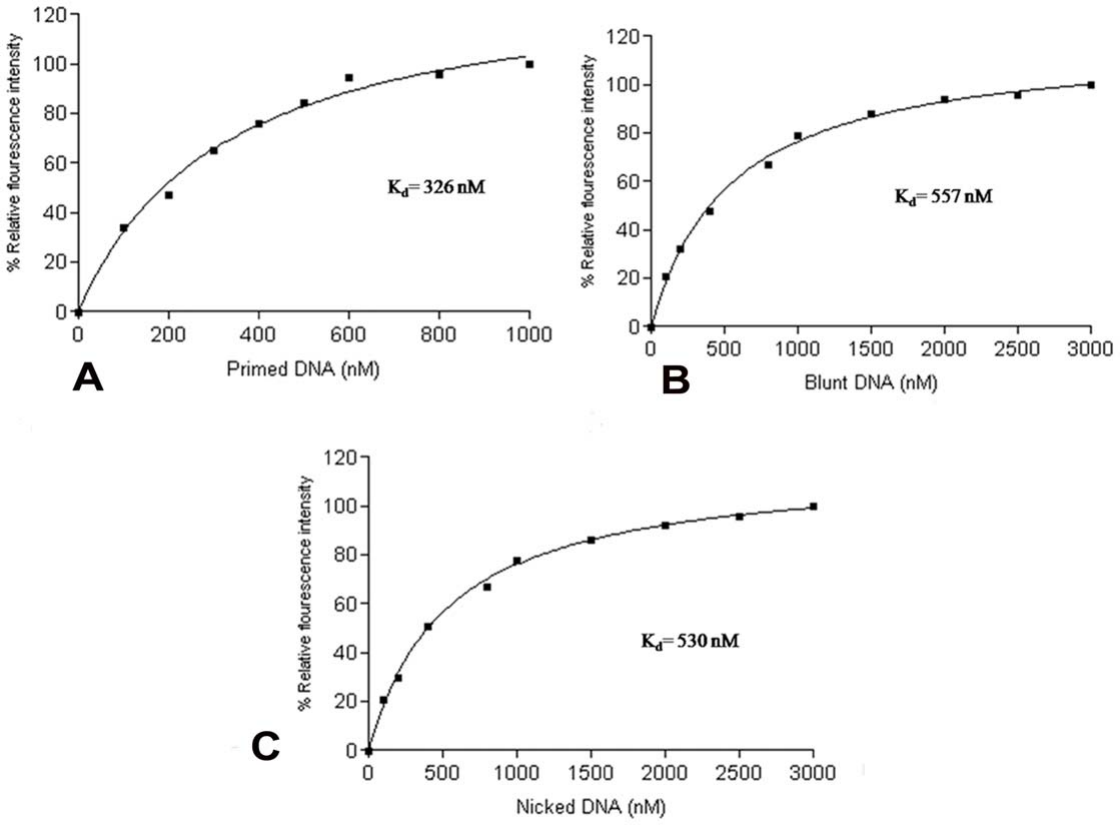
Plot of % relative fluorescence intensity (Y-axis) *versus* the respective concentrations (nM) of the DNA substrates (X-axis). The substrates were titrated against the labeled clamp protein, Mtbβ-Clamp^OG^. (**A**) Titration with a primed template (18/28), (**B**) titration against a blunt template (28/28) and (**C**) titration against a nicked DNA template (28/28). The apparent K_d_ values are shown in each plot.

Mtbβ-Clamp was cloned and purified by Ni-affinity chromatography. The DNA LigaseA (MtbLigA) and the γ-subunit of the DNA clamp loader complex of *M. tuberculosis* were also cloned and purified. The beta clamp is known to bind to DNA by encircling it and sliding along the duplex to help complete the DNA replication. Electrophoretic gel shift assays were initially carried out to probe interactions between the Mtbβ-Clamp and 18/18-mer blunt duplex, 18/18-mer nicked duplex, and 18/28-mer 3′ primed radio labeled template respectively. But no shift was detectable when this assay was used. Subsequently a more sensitive fluorescence based assay was performed. In this assay the Mtbβ-Clamp was labeled with a flourophore *viz.* OregonGreen488 maleimide (Molecular Probes, Inc., Eugene, OR) and called Mtbβ-clamp^OG^. This was carried out following the procedures described earlier [14]. The Mtbβ-clamp^OG^ was titrated with increasing concentration of dsDNA, nicked DNA and primed DNA respectively. The change in the fluorescence intensity was measured. The binding curves show saturation and therefore complete DNA-β-clamp complex formation occurs. The DNA binding assay was used to quanitate the affinity between the Mtbβ-Clamp and a short synthetic blunt DNA duplex, nicked DNA duplex and primed DNA duplex respectively. The results show an apparent K_d_ value of 557 nM for blunt end, 530 nM for nicked DNA and 326 nM for primed DNA respectively (**Figure 1**). These results suggest that there is not much difference in the binding involving nicked and blunt ds DNA. The ssDNA of the primed template, on the other hand, binds relatively strongly. This is probably because it involves binding to the peptide-binding groove as suggested earlier in the *E. coli* β-clamp [1]. Thus the Mtbβ-Clamp has sites for the binding of both ds DNA and ss DNA respectively.

### β-clamp interactions with the γ-subunit of the clamp loader and LigA respectively

**Figure 2:**
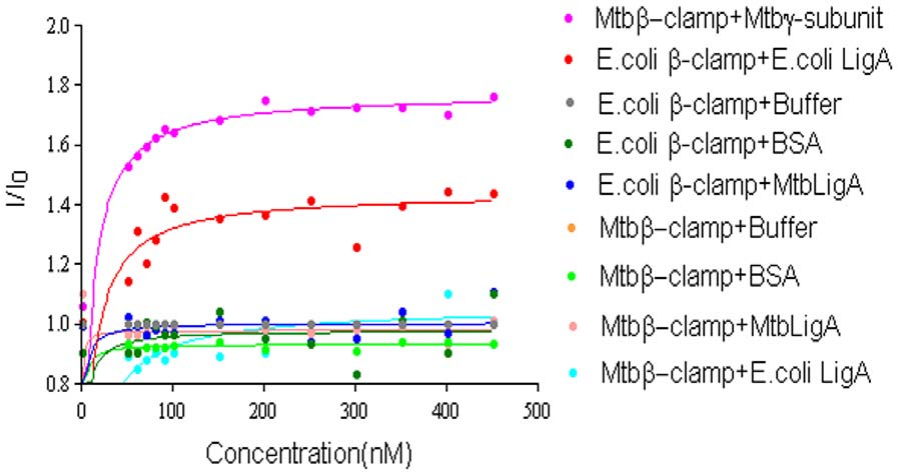
Interactions of the *M.tuberculosis* and *E. coli* β-Clamps with various proteins. Mtbβ-Clamp^OG^ (90 nM) was titrated with increasing concentration of the γ-clamp loader (Rv3721c) and LigA respectively (labeled in the figure). Interactions between the β-Clamp and LigA from *E. coli* was used as positive control where *E.coli* β-Clamp^OG^ (90 nM) was titrated with increasing concentration of *E.coli* LigA, and MtbLigA respectively. The *E. coli* LigA was also titrated against the Mtbβ-Clamp^OG^ to probe for their interactions too. BSA was used as a control for non-specific interactions. Changes to the relative fluorescence intensity were observed at λ_max_ 510.

Mtbβ-clamp^OG^ was titrated with varying concentrations of the DNA clamp loader gamma subunit to probe for their interactions. The results demonstrate that the γ-subunit exhibits relatively high affinity for the Mtbβ-Clamp with an apparent K_d_ of 23.8 nM (**Figure 2**). The latter result is in line with analogous experiments in *E. coli* where interactions of the *E. coli* β-clamp with the γ-subunit of the clamp loader complex has been demonstrated [5].

In the study involving interactions of LigA with the clamp, the control experiments involved the *E.coli* β-clamp and its LigA respectively. Earlier, interactions between these proteins in *E. coli* were demonstrated using radioactively labeled β- and native gel-shift assay [5]. In the present experiments, we demonstrated the interactions between these proteins through the titration of the *E. coli* β-clamp^OG^ with varying concentration of the *E. coli* LigA. The experiments show saturation and complete binding with apparent K_d_ of 42.5 nM. On the other hand, unlike in the *E. coli* case, MtbLigA does not exhibit affinity for the Mtbβ-Clamp under the assay conditions (**Figure 2**). Cross reactivity for the respective β-clamp and LigA from *E. coli* and *M. tuberculosis* was also probed. However no interactions between the *E. coli* LigA and the Mtbβ-Clamp or *vice versa* were observed.

We then tried to probe for effects of the Mtbβ-Clamp on the ligation activities of MtbLigA and carried out the ligation assay reported by us earlier [14] in the presence of increasing concentration of the Mtbβ-Clamp. No change was observed in the ligation activities of the MtbLigA in the presence of the clamp. This is unlike the human case where addition of PCNA increases the ligation activity of Human DNA ligase I [15]. The possibility of direct interactions between LigA and the β-Clamp in *M. tuberculosis* were therefore probed using additional approaches detailed in the next subsections.

### Radioactive gel shift assay to probe for potential Mtbβ-Clamp-MtbLigA interactions

**Figure 3:**
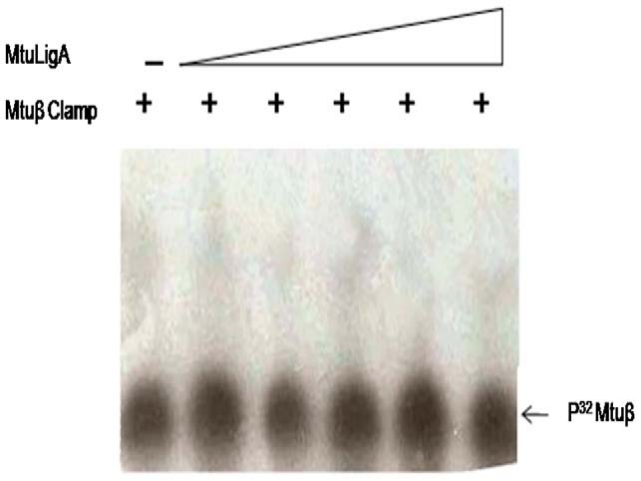
Radioactive Gel shift assay to probe for MtbLigA-Mtbβ-Clamp interactions. P^32^ labelled Mtbβ-Clamp (90 nM) was titrated against increasing concentration of MtbLigA. Samples were analysed on 6% Native PAGE. Shifts were analysed by autoradiography. No interaction could be detected between the proteins.

Radioactive gel shift assays were used as a sensitive alternate approach to probe for interactions between the two proteins. The possibility of direct interactions between the Mtbβ-Clamp and MtbLigA were probed by using labeled β-clamp with P^32^ at its C-terminus where a kinase motif had been incorporated. Gel shift assays were performed under varying conditions where different concentrations of MtbLigA were used. No shift was observed in any of the experiments and supports that no direct interaction exists between the proteins (**Figure 3**).

### Yeast two hybrid cytotrap system for Mtbβ-Clamp-MtbLigA interactions

**Figure 4:**
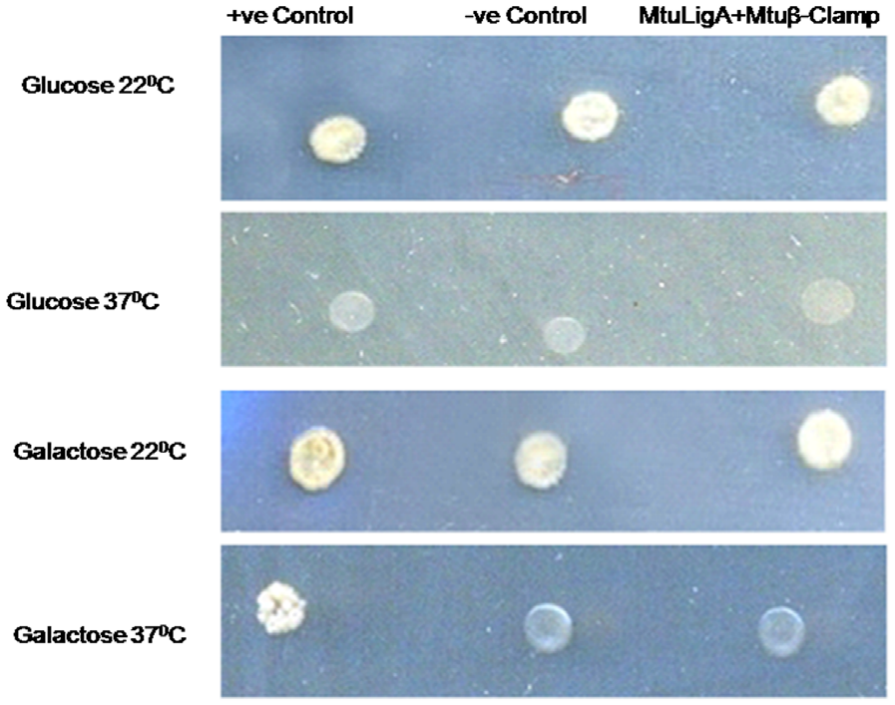
Yeast two-hybrid cyototrap experiments to probe for MtbLigA-Mtbβ-Clamp interactions. MtbLigA does not interact with the Mtbβ-Clamp in these experiments. No growth of yeast strain CDC25H bearing pSOS-*MtbLigA* and pMYR-Mtbβ-Clamp, at 37°C on galactose and on glucose media was observed. On the other hand, growth at 37°C was observed for the positive control on galactose media.

The yeast two-hybrid technique was used to probe for interactions between the proteins employing the commercially available kit supplied by *M/s Stratagene*. To probe for the interactions between Mtbβ-Clamp and MtbLigA, both genes were cloned into the two different yeast vectors pSOS and pMYR respectively. Both clones were co-transformed into the yeast cdc25H strain and grown in glucose and galactose containing media at 25°C and 37°C temperatures. The positive control was provided by the Stratagene Cytotrap kit. No growth was observed at 37°C in galactose containing media suggesting that Mtbβ-Clamp and MtbLigA do not interact (**Figure 4**). The results do not support the interactions of the Mtbβ-Clamp and MtbLigA under the *in vivo* conditions.

### Beta clamp crystal structure

**Figure 5:**
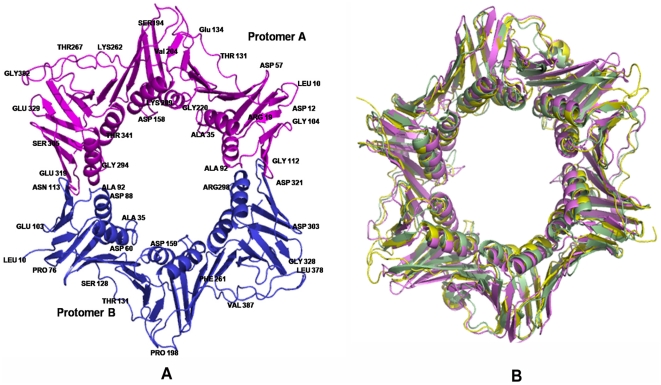
Crystal structure of the Mtbβ-Clamp and its superposition with other known structures of bacterial clamps. (**A**) The two chains that form the dimer have been coloured ‘blue’ and ‘pink’ respectively and are in cartoon representation. Selected residues have been labeled for clarity (**B**) Cartoon representation of the superposition of known structures of bacterial clamps *viz*. Mtbβ-Clamp, *yellow*; *E. coli* (PDB: 2POL), *pink*; *S. pyogenes* (PDB: 1AVT), *green*. This and the subsequent figures were made using PYMOL.

The structure was solved using molecular replacement (MR) techniques employing the structure of the *E. coli* clamp as the model for the calculations. The electron density maps were of good quality and permitted the tracing of the molecule. The final structure consists of two chains (**Figure 5a**). Chain A contains residues from 8 to 402 while Chain B contains residues from 10 to 402 respectively. Each protomer contains 3 distinct domains and the topology and secondary structure of these domains are similar to each other. Each domain contains a pair of four-stranded antiparallel β-sheets that bracket two antiparallel α-helices. Not unexpectedly, the structure is similar to that of other clamps from bacteria like the *E.coli* and *S. pyogenes* with minor variations (**Figure 5b**). While the manuscript was being prepared another group reported the structure of the Mtbβ-Clamp grown under different conditions and in the monoclinic P2_1_ space group [16]. The structural details that have been highlighted in the earlier report will be avoided here to avoid repetitive descriptions.

### Dimeric interface

**Figure 6:**
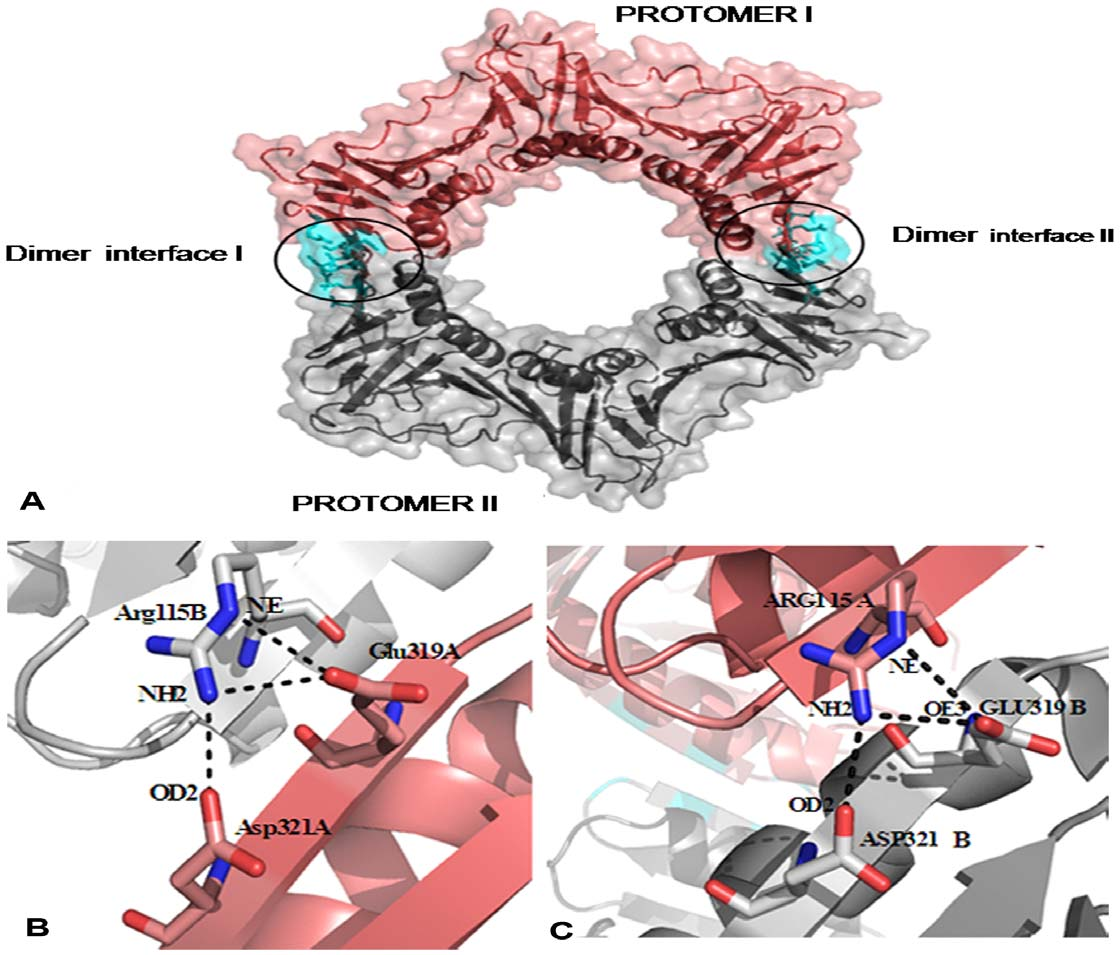
Interactions at the dimeric interface in the Mtbβ-Clamp. The two protomers are distinctly colored for clarity with the *van der Waals* surface overlaid on the cartoon representation (*top*). The respective interfaces are depicted in *cyan*. The close up of the two respective interfaces (*below left & right respectively*) depict the salt-bridges by *black* dotted lines. Some of the interacting residues are labeled.

There are two independent subunits in the asymmetric unit, which form a dimer (**Figure 6a**). The subunits of the dimer are held together by both hydrophobic and hydrophilic interactions. The inter-subunit interactions are listed in **Table 1** and highlight the extensive association between the subunits of the dimer. There are 21 polar interactions less than 3.6 Å and 6 salt bridges that stabilize the association of the Mtbβ-Clamp. The inter-subunit interactions in other beta clamps are also stabilized partly through salt bridges and hydrogen bonds (**Figure 6b**). The interaction details at the dimeric interface were calculated using the PISA server [17]. A total of 31 residues (7.9%) of both A and B chains form part of the intersubunit interface.

**Table 1:** Polar inter-subunit interactions in the Mtbβ-Clamp dimer.

Atoms in subunit A	Atoms in Subunit B	Distance (Å)
GLU319[N]	ARG115[O]	2.9
ARG106[NH2]	VAL315[O]	2.7
SER117[N]	ARG317[O]	2.8
ARG115[NH2]	GLU319[OE2]	3.3
ARG115[NE]	GLU319[OE2]	3.4
ARG115[N]	GLU319[O]	3.0
ARG115[NH2]	ASP321[OD2]	2.8
ASN113[ND2]	ASP321[O]	2.9
ASN113[O]	ASP321[N]	3.6
ARG115[O]	GLU319[N]	2.9
ASP314[O]	ARG106[NH2]	3.5
VAL315[O]	ARG106[NH2]	2.6
ARG317[O]	SER117[N]	2.8
GLU319[OE2]	ARG115[NE]	3.3
GLU319[OE2]	ARG115[NH2]	3.2
GLU319[O]	ARG115[N]	3.0
ASP321[OD2]	ARG115[NH2]	2.8
ASP321[O]	ASN113[ND2]	2.8
ARG115 [NH2]	GLU319 [OE3]	3.3
ARG115 [NE]	GLU319 [OE2]	3.4
ARG115 [NH2]	ASP321 [OD2]	2.8
GLU319 [OE2]	ARG115 [NE]	3.3
GLU319 [OE2]	ARG115 [NH2]	3.1
ASP321 [OD2]	ARG115 [NH2]	2.8

### Central cavity and interactions with DNA

**Figure 7:**
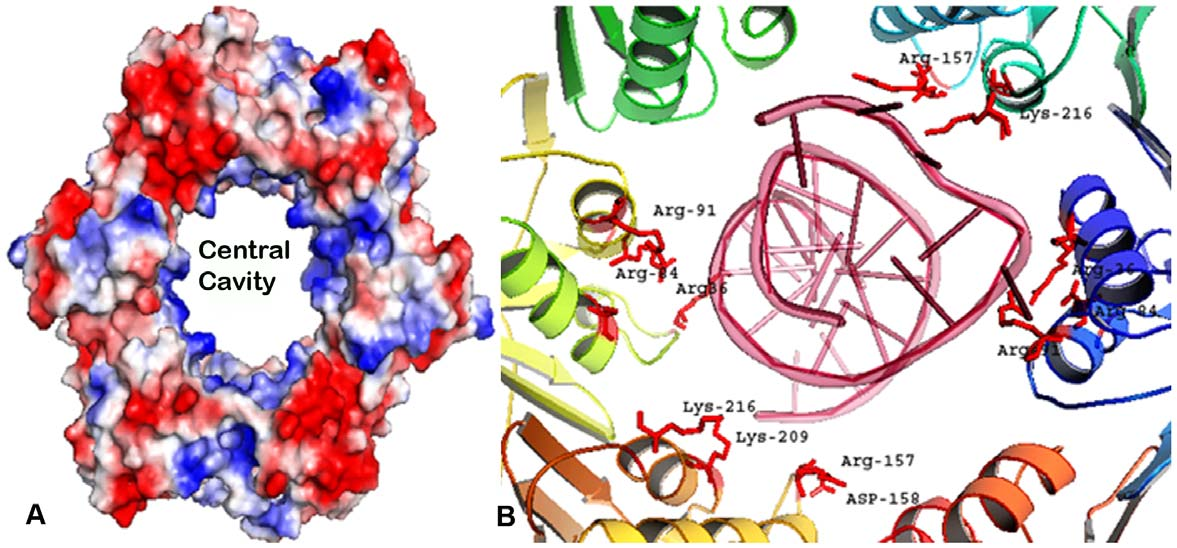
Electrostatic surface representation of the Mtbβ-Clamp and mapping of residues that interact with DNA. (**A**) *Red* indicates the negatively electrostatic potential; *white* indicates neutral and *blue* corresponds to positively charged regions. The central cavity is positively charged and is in line with its role in binding DNA. (**B**) Mapping of residues that interact with DNA in the *E. coli* β-clamp with that of corresponding ones in the Mtbβ-Clamp. Residues within 4 Å of the substrate after superposition of the respective crystal structures were identified and some of the conserved ones are labeled.

Electrostatic surface analysis shows that the inner part of the ring is electropositive in line with its DNA interacting functions (**Figure 7a**). The interior of the central hole in the Mtbβ-clamp is lined with basic residues, many of which (e.g., Arg 36, Arg 84, Arg 91,Arg 157, Lys 209, Lys 216, Lys 285 and Lys 384) are conserved between the *M. tuberculosis*, S. *pyogenes* and the *E. coli* proteins. The remainder of the protein is largely acidic; the protein has a calculated isoelectric point (pI) of 5.4. Most of the negatively charged patches on the surface of the *S. pyogenes* β subunit are found on the face of the clamp that is opposite to the predicted site of interaction with the clamp loader and polymerases and other proteins. The interactions of Mtbβ-clamp with DNA substrate was visualized by superimposing the *E. coli* beta clamp-DNA complex (3BEP.pdb) onto the former. Interactions of the DNA less then 4.5 Å were mapped onto the Mtbβ-Clamp (**Figure 7b**) and show that these are largely similar.

### Protein-Protein interaction site

**Figure 8:**
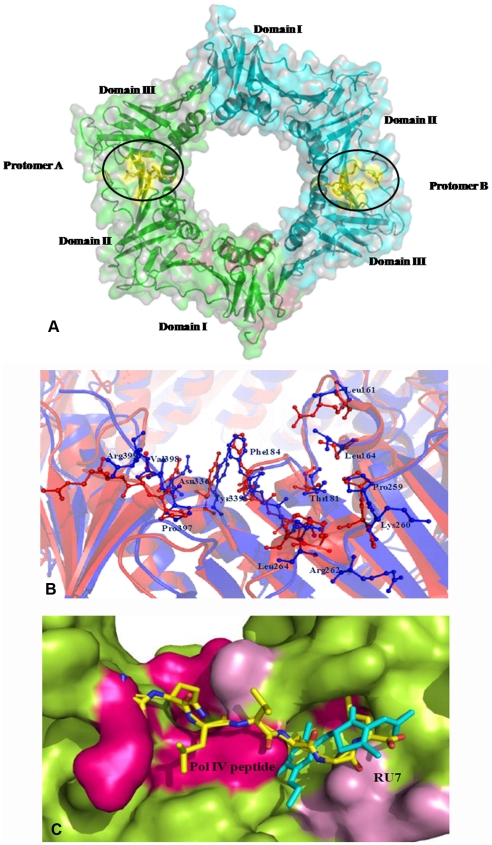
Peptide binding groove of the Mtbβ-Clamp, structural alignments and inhibitor interactions. (**A**) The hydrophobic groove, depicted in *yellow*, is present between domains II and III of each protomer. The residues of the respective hydrophobic grooves were found to be quite conserved among bacterial β-clamps. (**B**) Structural alignment of the peptide binding groove from the respective crystal structures of the Mtbβ-clamp (*blue*) and *E. coli* β-clamp (*red*). The Mtbβ-Clamp residues are numbered. (**C**) Superposition of the Mtbβ-Clamp crystal structure onto that of the *E. coli* β-clamp -RU7 inhibitor complex (PDB: 3D1G). The surface of the Mtbβ-clamp is colored *green* while the peptide binding pocket of Mtbβ-Clamp is colored according to the sequence conservation between Mtbβ-clamp and *E. coli* β-clamp; *dark pink* represent identical residues while *light pink* represents homologous residues. The RU7 inhibitor is depicted in *cyan* stick representation. The Pol IV peptide (yellow, stick representation) from its crystal structure complex with the *E. coli* clamp (PDB: 3D1E) is also shown. The inhibitor mainly interacts with subsite 1 of the peptide binding site.

The DNA beta clamp interacts with number of proteins and recruits them during the course of its functions [5]. These proteins include DNA polymerases, clamp loaders, replication initiation factor *etc* [18]–[19]. Proteins that bind to the bacterial β- clamp contain a five or six- residue consensus sequence, QL[S/D] LF and QLxLx [LF] [20]. A multiple sequence alignment of β-Clamp and LigA from various sources with focus on the interacting sites are in **Figures S1 & S2** respectively Crystal structures of the *E.coli* β-clamp with components of polymerases and the clamp loader revealed that the beta clamp contains a hydrophobic groove, which interacts with different proteins. This hydrophobic groove is present between domains II and III of each protomer (**Figure 8a**). In this context the structures of the *E. coli* β-clamp bound to the δ clamp loader subunit [21] and *E. coli* beta clamp and DNA polymerase IV peptide complex [22] are available. We accordingly mapped the residues of the hydrophobic groove identified in the *E. coli* clamp structure onto the Mtbβ-Clamp (**Figure 8b**). Superimposition of the respective structures of the *E. coli* beta clamp with PolIII (PDB: 3D1E), PolII (PDB: 3D1F), and PolIV peptides (PDB: 1OK7) and with δ clamp loader subunit onto the Mtbβ-Clamp were carried out. Comparison of the β-clamp sequences shows that residues that bind to the Pol III peptide are well conserved. Superposition of residues that form the protein-binding pocket of *E. coli* β-clamp (PDB: 2POL), clamp from *S. pyogenes* (PDB: 2AVT) and the Mtbβ-Clamp results in only minor deviations between the corresponding α-carbon atoms. The peptide-binding site itself has two subsites as reported earlier [10]. The structure of the peptide-β clamp complexes and sequence alignment of bacterial β-clamps suggests a consensus sequence for the peptide-binding pocket of bacterial clamps.

The beta-sliding clamp is recently attracting attention as a potential pharmaceutical target because it is essential for cell viability and shares little sequence homology with the eukaryotic PCNA clamp. One group has reported the co-crystal structure of a small molecule inhibitor that binds to protein binding domain of the *E. coli* β-clamp (**Figure 8c**). Such inhibitors can potentially disrupt the binding of other proteins to the beta clamp as shown earlier [1]. The binding of the RU7 compound to the *E. coli* beta clamp is able to inhibit its interactions with PolIII and also inhibits the DNA synthesis by 5–50 times [10]. The comparative analysis with the present crystal structure shows that the inhibitor binding site is largely conserved in the Mtbβ-clamp also. Overall the comparative structural analysis shows that DNA binding and peptide binding sites are relatively well conserved in the Mtbβ-clamp compared to the lower sequence identity between the beta clamps.

## Discussion

We started characterizing the Mtbβ-clamp–MtbLigA interactions assuming that these proteins will interact strongly as shown earlier *eg.* between human PCNA and Ligase-I and for the *E. coli* LigA and its clamp respectively [5]–[6]. Our intention was to attempt the structural characterization of these predicted interactions as no report is known involving the bacterial clamp. Admittedly, our notion that the bacterial LigA ought to interact with the bacterial β-clamp was predicated on a single earlier report [5] where the results were based on the protein gel-shift experiments involving the *E. coli* proteins. To the best of our knowledge, no subsequent report involving interactions between these bacterial proteins has been reported by others while several studies, as mentioned earlier, have characterized the interactions between the human/eukaryotic homologs. Our control experiments involving the *E. coli* proteins confirm the interactions. The lack of direct *in vitro* interactions between the proteins in *M. tuberculosis* was unanticipated and points to more basic differences in the interactions between these proteins in bacterial and eukaryotic systems. Multiple sequence analysis of the β-clamp proteins from *M. tuberculosis*, *E. coli* and other sources does not reveal any obvious differences in the peptide binding site of the clamp (**Figure S1**). On the other hand, sequence alignments of LigA, especially around motifs thought to be important for protein-protein interactions (**Figure S2**), suggests that there are distinct conserved differences in Mycobacteria in these motifs. Two of them *viz.* L–F-Y and Q–L-F/Y are situated in the adenylation domain while the third motif KL-KA–L is located in the BRCT domain situated at the C-terminus of LigA. The relevance of these differences in protein-protein interactions remains to be seen. In this context our ongoing work in identifying the determinants of the interaction between the β- and the LigA in the *E. coli* system through mutations and other strategies should be interesting.

*M. tuberculosis/mycobacteria* exhibit novel variations in the DNA repair and replication pathways. The non-homologous end joining (NHEJ) DNA repair pathway found in some bacteria was first identified in *M. tuberculosis* recently [23]. This repair pathway is not present in most bacteria like *E. coli*. Mismatch repair proteins like the MutS have not been identified in mycobacteria despite the genome sequence being available [24]. The principal replicative ligase LigA takes part in homologous recombination (HR) processes, as in other bacteria, and additionally in NHEJ repair in mycobacteria [25]. The lack of direct interactions reported here between MtbLigA and Mtbβ-clamp may be another example of the variations of the pathways in mycobacteria responsible for DNA metabolism. The characterization of the Mtbβ-clamp shows that it is an active protein with specific affinity for DNA and that it interacts with other partners. MtbLigA that was used in the experiments is also active and exhibits all the activities associated with LigA *in vitro* [13], [26]. DNA, as a mediator of MtbLigA and Mtbβ-clamp interactions, is ruled out by the experiments performed in the presence of DNA. The presence of the Mtbβ-clamp alone has no effect on the *in vitro* ligase activities of MtbLigA in contrast to other reports which show that PCNA modulates the activity of the DNA ligase I [15]. It seems very unlikely that LigA and the β-clamp do not interact at all in mycobacteria/*M. tuberculosis*; especially given that the ligase plays an essential role in many DNA transactions including the final step of nick sealing at the end of several DNA repair pathways. The results therefore suggest that other as-yet-unidentified factors/mechanisms may mediate interactions between these proteins and DNA.

Until the recent experiments in *E. coli* [1], it was thought that the β-clamp does not exhibit specific affinity for DNA. We carried out affinity experiments with the same DNA substrates *viz.* primed DNA and blunt dsDNA, as used earlier in *E. coli* to rule out variations in affinity because of the substrate. Additionally we used nicked DNA substrate, partly because of our intent to search for favorable *in vitro* conditions for interactions between the MtbLigA and the Mtbβ-clamp. We find that the mycobacterial clamp also has specific affinity for DNA and exhibits about 1.8 times higher affinity for primed DNA compared to nicked and dsDNA respectively. It is not surprising that the affinity for nicked and dsDNA are similar because the clamp has no known nick sensing activity. The *E. coli* clamp, on the other hand, exhibits stronger relative affinity for primed DNA. In fact its affinity for primed DNA is about 4 times higher than for blunt dsDNA. However the affinity of both homologs for blunt dsDNA is similar with K_d_ 453 and 557 nM for the *E. coli* and *M. tuberculosis* clamps respectively.

The structural conservation of the Mtbβ-Clamp as seen in the crystal structures is not surprising, especially for a protein performing an important role in various pathways involving DNA. What is surprising is that it does not exhibit interactions with LigA unlike as described earlier *eg.* in *E. coli* and human systems. The comparative studies demonstrate that the peptide and DNA recognition region(s) in the Mtbβ-Clamp is well conserved compared to other clamps and no obvious differences could be detected in these sites to ascribe the lack of interactions of the Mtbβ-Clamp with the LigA. *In vivo* studies are necessary to probe and identify the predicted unknown factors that should mediate Mtbβ-Clamp- LigA interactions. The latter studies should afford insights to the conjectured differences in the DNA repair pathways in mycobacteria compared to other bacteria in which these proteins are involved. The present studies should additionally help in the characterization of the β-clamp as a novel therapeutic target.

## Materials and Methods

### Cloning and purification of proteins

The sequence analysis of Mtbβ-Clamp (Rv0002) was carried out using ClustalW and available sequences of some characterized bacterial clamps (**Figure S1**). The gene corresponding to Rv0002 is 1209 bp long and was amplified by PCR by using the sense 5′-TTT**GGATCC**ATGGACGCGGCTACGACAAGA-3′ and antisense 5′-AAT**AAGCTT**TCCCGGCAACCGAACTGGCATCAA-3′ primer set designed for C-terminal 6X his-tagged protein. It was cloned into pET23a between BamHI and HindIII and the resulting construct was transformed into *E. coli* BL21 (DE3). Mtbβ-Clamp cultures were grown at 30°C temperature. Protein expression was induced at an OD_600_ ∼0.4–0.6 by the addition of 0.5 mM IPTG followed by further growth for 8–10 hrs. Culture was harvested at 7000 rpm and resuspended in a buffer containing 50 mM Tris 8.0, 200 mM NaCl and 10 mM Imidazole. Cells were lysed by sonication and purified by affinity chromatography employing a Ni-IDA column (GE Healthcare). Protein fractions were pooled, precipitated using ammonium sulfate (40%) and re-suspended in 50 mM Tris-HCl pH 8.0, 50 mM NaCl, and 5 mM EDTA. Trace contaminants were removed by using a Superdex S-200 10/300GL column (GE Healthcare).

The γ-subunit of DNA clamp loader (Rv3721c), the LigA (Rv3014c) and the *E. coli* β-clamp were cloned into the pQE60, pET21d and pET23a expression vectors with 6X-His tag respectively.

The primer pairs for the same are:

*Forward primer LigA*: 5′-CTGGGTACCGCCATGGCAGACTCGGATTTA3′

*Reverse primer LigA*: 5′-TGTAAGCTTCGTTCGTGAGGCGGGTCCGTC-3′

*Forward primer* γ-subunit: 5′-AAT**CCATGG**TGGCTCTCTACCGAAAGTACCGA-3′

*Reverse primer* γ-subunit: 5′-ATT**GGATCC**AGCTGCAGCCGACGGACG-3′

*Forward primer E.coli β*: 5′-CCA **GGATCC**ATGAAATTTACCGTAGAACG-3′

*Reverse primer E.coli β* : 5′-GTG**AAGCTT**CAGTCTCATGTTCATTACAAC-3′

MtbLigA and *E. coli* β-clamp were overexpressed in BL21 (DE3) cells while the Mtb-γ-subunit was overexpressed in TG1 cells respectively. The proteins were purified by affinity chromatography employing a Ni^++^-IDA column following similar procedures to that described above for the Mtbβ-Clamp.

### Crystallization and structure solution

The hanging drop method was used for crystallizations and the plates were incubated at 295 K. Mtbβ-Clamp native crystals were obtained by mixing 2 µL of 10–12 mg/mL protein and 2 µL of reservoir solution containing 9% Isopropanol, 12% PEG4K and 0.1 M Na HEPES pH 7.0. A single crystal was mounted in a nylon loop and flash-cooled in a nitrogen stream at 100 K after a brief soak in the reservoir solution supplemented with 25% glycerol. Diffraction data were collected using a Rigaku MICROMAX-007 X-ray generator coupled to a MAR345-dtb image plate detector and processed using MOSFLM and programs implemented in the CCP4 package [27].

### Structure solution and refinement

Amino acid sequence alignment showed 29% identity with the *E. coli* β-clamp (PDB; 2POL). Therefore, a search model derived from this structure was used in the molecular replacement calculations using the program PHASER [28] implemented in the CCP4 package [27]. The best solution was obtained in the space group C222_1_ and the packing corresponding to the solution showed that there were no steric clashes between symmetry related molecules. The transformed model was subjected to rigid body refinement using REFMAC5 [29]. The 2*F*_o_−*F*_c_ and *F*_o_−*F*_c_ electron density maps were visualized using the program COOT [30] that was used for model building (**Figure S3**). The crystallographic *R*-factor and *R*-free were monitored at each stage. The refined model of Mtbβ-Clamp contains two chains. The final refinement statistics are given in **Table 2**.

**Table 2:** Data collection and refinement statistics.

*Data Collection*	
Data set	Native Mtbβ-Clamp
Space group	C222_1_
Cell dimensions	a = 72.7,b = 234.9,c = 125.1
Wavelength (Å)	1.5418
Resolution	41.1-3.0 (3.16-3.0)
Unique reflections	21,391 (3,081)
Completeness (%)	99.9 (100)
Redundancy	3.6 (3.6)
*R_sym_	8.7 (64.8)
I/σI	12.9 (1.9)
*Refinement*	
Resolution range	41.1-3.0 (3.16-3.0)
Reflections	21,391
*Total no. of Atoms*	
Protein	5813
Water	15
R_factor_ (%)	21
R_free_ (%)	25
*r.m.s.d.*	
Bond (Å)	0.010
Angle (Å)	1.426
*Ramachandran plot*	
Most favored and additional allowed regions	96.7%
Disallowed region	3.3%

* R_sym_ = ΣhΣi|Ih,i− Ih|/ΣhΣiIh,i, where Ih is the mean intensity of the i observations of symmetry related reflections of h. R_factor_ = Σ|Fobs−Fcalc|/ΣFobs, where Fobs = FP, and Fcalc is the calculated protein structure factor from the atomic model (R_free_ was calculated with 5.0% of the reflections).Values in parentheses correspond to the highest resolution shell.

The geometry of the refined models was checked using PROCHECK [31]. Τhe structural superimposition and r.m.s.d. calculations were carried out using PROFIT (http://www.bioinf.org.uk/software/profit). 96.7% of all residues are in allowed regions of the Ramachandran plot. Residues in the disallowed regions were found to be mostly in the flexible, interconnecting loop regions and exhibit relatively weaker electron density. Programs from the CCP4 package were used to carry out the B-factor and other analyses. Interface interactions were calculated using the PISA server (http://www.ebi.ac.uk/msd-srv/prot_int/cgi-bin/piserver). Figures were prepared using the programs CCP4MG [32] and Pymol [33].

### DNA binding activity of the Mtbβ-clamp

Mtbβ-clamp was labeled with OregonGreen488 maleimide (Molecular Probes, Inc., Eugene, OR) to form Mtbβ^OG^-Clamp following similar procedures as reported for the *E. coli* clamp [5]. Briefly 500 µM protein was incubated with 1 mM of OregonGreen488 maleimide at 4°C for overnight and then purified by size exclusion column Superdex-200 10/300 GL. The following oligonucleotides were used to construct 18/18-mer blunt duplex and 18/28-mer 3′ primed template and Nicked Duplex template: 5′-CCCATCGTATAGCAAGGG-3′ (18-mer primer), 5′-CCCTTGCTATACGATGGG-3′ (18-mer template), 5′-TTTTTTTTTTCCCTTGCTATACGATGGG-3′ (28-mer template), and 5′-AAAAAAAAAA-3′. DNA titrations contained Mtbβ^OG^-Clamp (200 or 500 nM) in 60 µl of 20 mM Tris-Cl (pH 7.5), 1 mM DTT, 0.2 mM EDTA, and 40 mM NaCl. Reactions were equilibrated at 22°C for 10 min and then transferred into a 3×3 mm cuvette. Fluorescence emission spectra were recorded from 500 to 630 nm (excitation at 490 nm); emission at 517 nm was used for analysis.

### Fluorescence based assay for probing interactions of Mtbβ-clamp with MtbligA and the γ-clamp loader respectively

Mtbβ^OG^-Clamp was used in the assays using procedures similar to those reported earlier for the *E.coli* clamp [34]. Reactions contained Mtbβ^OG^-Clamp (50 or 100 nM) in 60 µl 20 mM Tris-Cl (pH 7.5), 8 mM MgCl_2_, 1 mM DTT, 0.2 mM EDTA, 50 mM NaCl, and 0.5 mM ATP. Titrations with MtbligA and the γ-clamp loader were performed. Reactions were equilibrated at 22°C for 10 min and then transferred into a 3×3 mm cuvette. Fluorescence emission spectra were recorded from 500 to 600 nm (excitation at 490 nm); emission at 510 nm was used for the analyses. The data were analyzed using *GraphPad Prism* for the determination of the apparent dissociation constant (K_d_) values using standard methods. The interactions between the β-clamp^OG^ and LigA in *E.coli* were used as the positive control while BSA was used as the control for non-specific interactions.

### Gel shift assays using radioactively labeled Mtbβ-Clamp

Interactions of the Mtbβ-Clamp with MtbLigA were probed using gel shift assays. The Mtbβ-clamp subunit, modified at the C terminus to incorporate a protein kinase motif, was labeled with [γ-^32^P] ATP by protein kinase similar to procedures described earlier [7]. Reactions (15 µl) contained 20 mM Tris-Cl (pH 7.5), 0.1 mM EDTA, 4% glycerol, 50 µg/ml BSA, 100 mM NaCl, 5 mM DTT and 90 nM [^32^P] Mtbβ-Clamp. Different concentrations of MtbLigA were respectively incubated on ice with [^32^P] Mtbβ-Clamp for 4 min. The reactions were stopped by adding 1 mM EDTA. 5 µl of reaction was loaded on a native 4% polyacrylamide gel. Electrophoresis was performed by using TBE buffer (90 mM Tris-Cl/64.6 mM Boric acid/2.5 mM EDTA, pH 8.3) at 17 mA and 22°C. Gels were dried and detection of radioactive Mtbβ-Clamp was performed by exposing the X-ray films (Molecular Dynamics).

### Yeast Two–Hybrid CytoTrap system

The Yeast two hybrid CyotoTrap system (M/s Stratagene) was used to probe for MtbLigA and Mtbβ-clamp interactions *in vivo*. MtbLigA was cloned as the bait into the pSOS vector (fusion with hSOS) between the NcoI restriction sites. The Mtbβ-clamp was cloned as the target into pMyr (src myristylation signal that targets and anchors the protein to the cell membrane with the gene product extruding into the cytoplasm) between EcoRI and BamHI restriction sites. Both the constructs were co-transformed into the cdc25H yeast strain. After 2 days of growth under nonselective conditions, the screening plates were replica plated onto galactose medium and selected for protein-protein interactions at 37°C. Clones from colonies that exhibit galactose-dependent growth at 37°C were verified for a true interaction by showing bait-dependent growth on galactose medium at 37°C.

### DNA ligation activity

*In vitro* assays for ligase activity were performed using a double-stranded 40 bp DNA substrate carrying a single-strand nick between bases 22 and 23 as reported earlier [11]–[13]. Briefly, the substrate was created in TE buffer by annealing 22- and 18 mer DNA complementary strands to a 40 mer (5′-ATG TCC AGT GAT CCA GCT AAG GTA CGA GTC TAT GTC CAG G-3′). At the 5′ end, the 18 mer was radiolabeled with [γ-^32^P] ATP (3000 Ci/mmol, Board of Radiation and Isotope Technology, Mumbai).

Reaction mixtures (15 µl) containing 50 mM, Tris–HCl, pH 8.0, 5 mM DTT, 10 mM MgCl2, 50 µM NAD^+^, 2 pmol of ^32^P-labeled nicked duplex DNA substrate and increasing concentration of Mtbβ-Clamp were incubated for 1 h at 25°C. Subsequently, they were quenched with formamide and EDTA. The products were resolved electrophoretically on a 15% polyacrylamide gel containing 8 M urea in TBE Buffer. Autoradiograms were developed and ligation extents were measured using Image Master 1D Elite software (GE Health care).

### Accession codes

The co-ordinates and structure factors have been submitted to the *Protein Data Bank* (http://www.rcsb.org) with the accession number 3RB9.

## Supporting Information

Figure S1 Sequence alignment of the beta-clamp proteins from *M. tuberculosis, E. coli, S. pyogene & T. maritima* respectively. The conserved residues are highlighted with red boxes. The residues marked with a red ‘star’ are involved in binding of a small molecule inhibitor RU7 in the peptide binding pocket of the *E.coli* beta clamp. The compound interacts with V247, P242, R152, R246, M362, T172 of *E.coli* beta clamp (O'Donnell *et al.*, 2008). The marked residues are conserved in *M. tuberculosis*. Alignments were carried out using ClustalW and the figure was generated using ESPript2.2.
(DOC)

Figure S2 Sequence alignment of the respective NAD^+^ -dependent DNA ligase proteins from different eubacterial species. Sequence snippets A, B and C shown below are around the three motifs suggested to be important for the interactions of LigA/LigI with co-proteins in other systems (See References below). The analysis reveals that mycobacteria exhibit definite differences (highlighted in ‘yellow’) in these motifs. The alignments were carried out using ClustalW.
(DOC)

Figure S3 2Fo-Fc electron density map (blue mesh) contoured at 1σ around (**A**) Residues from 256–263 of Chain A and (**B**) Residues 184–186 (residues of the hydrophobic peptide binding groove).
(DOC)
