# Supplementary material for: M. tuberculosis Sliding β-Clamp Does Not Interact Directly with the NAD+ -Dependent DNA Ligase
Source: PLoS One. 2012 Apr 24;7(4):e35702. doi: 10.1371/journal.pone.0035702 (PMC3335792; doi:10.1371/journal.pone.0035702)

Vandana Kukshal *et al*., 2012

**Figure S1.** Sequence alignment of the beta-clamp proteins from M. tuberculosis, E. coli, S. pyogene & T. maritima respectively. The conserved residues are highlighted with red boxes. The residues marked with a red ‘star’ are involved in binding of a small molecule inhibitor RU7 in the peptide binding pocket of the *E.coli* beta clamp. The compound interacts with V247, P242, R152, R246, M362, T172 of *E.coli* beta clamp (O’Donnell *et al*., 2008). The marked residues are conserved in M. tuberculosis. Alignments were carried out using ClustalW and the figure was generated using ESPript2.2.


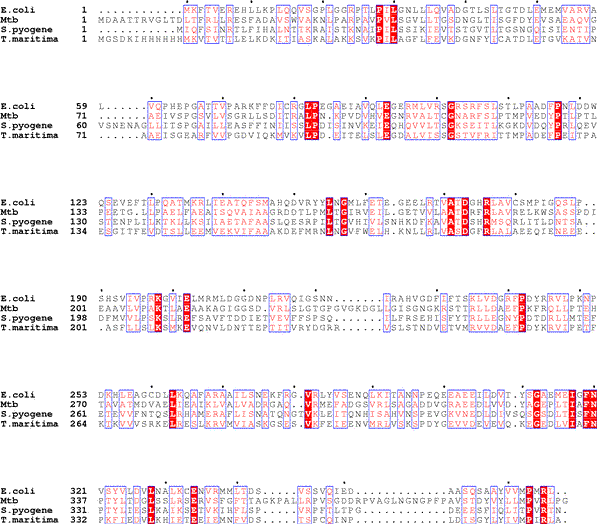

Supplement: Figure S1 — Sequence alignment of the beta-clamp proteins from M. tuberculosis, E. coli, S. pyogene & T. maritima respectively. The conserved residues are highlighted with red boxes. The residues marked with a red ‘star’ are involved in binding of a small molecule inhibitor RU7 in the peptide binding pocket of the E.coli beta clamp. The compound interacts with V247, P242, R152, R246, M362, T172 of E.coli beta clamp (O'Donnell et al., 2008). The marked residues are conserved in M. tuberculosis. Alignments were carried out using ClustalW and the figure was generated using ESPript2.2. (DOC) [file pone.0035702.s001.doc]
