# Supplementary material for: M. tuberculosis Sliding β-Clamp Does Not Interact Directly with the NAD+ -Dependent DNA Ligase
Source: PLoS One. 2012 Apr 24;7(4):e35702. doi: 10.1371/journal.pone.0035702 (PMC3335792; doi:10.1371/journal.pone.0035702)
Supplement: Figure S2 — Sequence alignment of the respective NAD+ -dependent DNA ligase proteins from different eubacterial species. Sequence snippets A, B and C shown below are around the three motifs suggested to be important for the interactions of LigA/LigI with co-proteins in other systems (See References below). The analysis reveals that mycobacteria exhibit definite differences (highlighted in ‘yellow’) in these motifs. The alignments were carried out using ClustalW. (DOC) [file pone.0035702.s002.doc]

Vandana Kukshal *et al*., 2012

**Figure S2.** Sequence alignment of the respective NAD^+^ -dependent DNA ligase proteins from different eubacterial species. Sequence snippets A, B and C shown below are around the three motifs suggested to be important for the interactions of LigA/LigI with co-proteins in other systems (See References below). The analysis reveals that mycobacteria exhibit definite differences (highlighted in ‘yellow’) in these motifs. The alignments were carried out using ClustalW.

| *A.* | **L--F-Y**  *B.amyloliquefaciens* LDIFVYSIAE----LDEMGVETQSQGLDFLDELGFKTNQERKQCATIDEVI 264  *B.subtilis* LDIFVYSIAE----LDEMGVETQSQGLDFLDELGFKTNQERKKCGSIEEVI 263  *S.aureus* LSVFIYSVND----FTDFNARSQSEALDELDKLGFTTNKNRARVNNIDGVL 260  *S.pneumoniae* LATFLYQEAS----PSTR--DSQEKGLKYLEQLGFVVNPKRILAENIDEIW 255  *S.pyogenes* LATFLYQEAS----PTAR--NQQNEVLAELADLGFSVNPYYQLTSSMDEIW 255  *S.enterica* LTFFCYGVGILE---GGELPDTHLGRLLQFKAWGLPVSDRVTLCDSPQAVL 267  *E.ceaebacterium* LTFFCYGVGLMD---GGELPASHYARLQQFKAWGLPVSDRVKRCTGSDEVL 272  *C.perfringens* LSAFFYDVGYK----EGAPFKTYMEMLNFIKTKGFPMDDYIRECTTLDEIQ 269  *C.botulinum* LSAFFYDVGYN----EGPEFKSYREMMNFIKNMGLPQDKYIKECTNMEEVE 267  *B.thuringiensis* LDAFWYDIAYS----EGITFETQAEMMTFLQEQGFKINPYFYVTDSIDVII 259  *T.filiformis* LRATFYALGLGLEES---GLKSQYELLLWLKEKGFPVEHGYEKALGAEGVE 268  *T.thermophilus* LRATFYALGLGLEEVEREGVATQFALLHWLKEKGFPVEHGYARAVGAEGVE 271  ***E.coli*** LTFFCYGVGVLE---GGELPDTHLGRLLQFKKWGLPVSDRVTLCESAEEVL 267  ***M.tuberculosis*** LRMICHGLGH----VEGFRPATLHQAYLALRAWGLPVSEHTTLATDLAGVR 277  *M.bovis* LRMICHGLGH----VEGFRPATLHQAYLALRAWGLPVSEHTTLATDLAGVR 277  *M.bovisBCGstr* LRMICHGLGH----VEGFRPATLHQAYLALRAWGLPVSEHTTLATDLAGVR 277  *M.ulcerans* LRMICHGLGH----TEGFRPATLHQAYLALQAWGLPVSQHTTLVADLAEVQ 265  *M.leprae* LRMICHGLGH----IEGFRPATQHQAYLALRDWGLPVSEHTTLVNDIAGVQ 277  *M.lepromatosis* LRMICHGLGH----IEGFRPATQHQAYLALRAWGLPVSDHTTLVNDIAGVQ 269  *M.rhodesiae* LRMICHGLGY----TEGFTPDTLHDTYFALKAWGLPVSDHTTRVAGLAAVE 265  *M.parascrofulaceum* LRMICHGIGR----TDGFAPASLHDAYTALKAWGLPVAEQTARVRGLAAVE 277  *M.smegmatis* LRMICHGIGY----TEGFTPASLHDAYRALGAWGLPVSEHTTKVSTVAEVA 285 |
| --- | --- |
| B. | **Q- -L-F/Y**  *B.amyloliquefaciens* EMIEELQAKRADLPYEIDGIVIKVDSLDQQEELGYTAKSPRWAIAYKFPA 314  *B.subtilis* TLIDELQAKRADLPYEIDGIVIKVDSLDQQEELGFTAKSPRWAIAYKFPA 313  *S.aureus* EYIEKWTSQRESLPYDIDGIVIKVNDLDQQDEMGFTQKSPRWAIAYKFPA 310  *S.pneumoniae* NFIQEVGQERENLPYDIDGVVIKVNDLASQEELGFTVKAPKWAVAYKFPA 305  *S.pyogenes* DFIKTIEAKRDQLAYDIDGVVIKVNSLAMQEELGFTVKAPRWAIAYKFPA 305  *S.enterica* DFYHNVEKDRPTLGFDIDGVVIKVNSLALQEQLGFVARAPRWAVAFKFPA 317  *E.ceaebacterium* KFYRQVEQERPTLGFDIDGVVIKVDSIEIQEQLGFVARAPRWATAFKFPA 322  *C.perfringens* KEIDYIRDIRFDLNYDIDGLVIAIDDIRTRDLLGYTVKFPKWAIAYKFEA 319  *C.botulinum* KEIEYIESIRGELDYDIDGAVIVVDDIKTREILGYTIKFPKWAIAYKFEA 317  *B.thuringiensis* EKLEEMKEVRPTLNWDIDGMVIKVNELHIREELGYTSKFPKWAIAYKFEA 309  *T.filiformis* EVYRRFLAQRHALPFEADGVVVKLDDLALWRELGYTARAPRFALAYKFPA 318  *T.thermophilus* AVYQDWLKKRRALPFEADGVVVKLDELALWRELGYTARAPRFAIAYKFPA 321  ***E.coli*** AFYHKVEEDRPTLGFDIDGVVIKVNSLAQQEQLGFVARAPRWAVAFKFPA 317  ***M.tuberculosis*** ERIDYWGEHRHEVDHEIDGVVVKVDEVALQRRLGSTSRAPRWAIAYKYPP 327  *M.bovis* ERIDYWGEHRHEVDHEIDGVVVKVDEVALQRRLGSTSRAPRWAIAYKYPP 327  *M.bovisBCGstr* ERIDYWGEHRHEVDHEIDGVVVKVDEVALQRRLGSTSRAPRWAIAYKYPP 327  *M.ulcerans* ARIDYWGEHRHEVDHEIDGVVVKVDDVALQRRLGSTSRAPRWAIAYKYPP 315  *M.leprae* GCIAYWAEHRHEVDHEIDGIVVKIDEVTLQRRLGSTSRAPRWAIAYKYLP 327  *M.lepromatosis* RRIAYWAEHRHEVDHEIDGIVVKIDELALQRRLGSTSRAPRWAIAYKYLP 319  *M.rhodesiae* EKITYWGERRHEIEHEIDGIVVKVDDFALQRRLGATSRTPRWAVAYKYPP 315  *M.parascrofulaceum* DRIGYWGEHRYEIDHEIDGVVVKVDDFALQRRLGTTSRAPRWAVAYKYPP 327  *M.smegmatis* ERIAYWGEHRHDVEHEIDGVVVKVDEVALQRRLGATSRAPRWAVAYKYPP 335 |
| C. | **KL-KA--L**  *B.amyloliquefaciens* KLEELSRNEAKTQIEALGGKLTGSVSKKTDLLIAGEAAGSKLTKAQELNI 655  *B.subtilis* KLEELSRNEAKAQIEALGGKLTGSVSKNTDLVIAGEAAGSKLTKAQELNI 654  *S.aureus* KLHQMTRNEASKWLASQGAKVTSSVTKNTDVVIAGEDAGSKLTKAQSLGI 650  *S.pneumoniae* KLERLKRSEAKSKLESLGAKVTGSVSKKTDLVVVGADAGSKLQKAQELGI 641  *S.pyogenes* KLNQLNRNEAKDKLEALGAKVTGSVSKKTDLVIAGSDAGSKLEKAKSLGI 641  *S.enterica* SLNQMSRDDAKERLVALGAKVAGSVSKKTDLVIAGEAAGSKLAKAQELGI 657  *E.ceaebacterium* SLSLMPRDEAKDRLAALGAKVSGSVSKKTDMVIAGEAAGSKLAKAQELGI 664  *C.perfringens* TLENYSRTSIKEKLESLGAKVSGSVSKKTDFVIAGEAAGSKYDKAKSLGV 653  *C.botulinum* SLNNYSRGEIKDKLQSLGAKVSSSVSKNTDYVLVGEKPGSKYEKAIELGV 651  *B.thuringiensis* KVS-RPRKEIEAFIKEHGGKASGSISKNTNYLVAGEAAGSKLAKAQSLGV 641  *T.filiformis* ELS-RPREEVKALLQRLGAKVTDSVSRKTSYLVVGENPGSKLEKARALGV 649  *T.thermophilus* ELS-RPREEVKALLRRLGAKVTDSVSRKTSYLVVGENPGSKLEKARALGV 652  ***E.coli*** SLSQMSRDDAKARLVELGAKVAGSVSKKTDLVIAGEAAGSKLAKAQELGI 657  ***M.tuberculosis*** SLTGFSRDDAKEAIVARGGKAAGSVSKKTNYVVAGDSPGSKYDKAVELGV 671  *M.bovis* SLTGFSRDDAKEAIVARGGKAAGSVSKKTNYVVAGDSPGSKYDKAVELGV 671  *M.bovisBCGstr* SLTGFSRDDAKEAIVARGGKAAGSVSKKTNYVVAGDSPGSKYDKAVELGV 671  *M.ulcerans* SLPGFSRDEAKEAIVTRGGKAAGSVSKKTSYVVAGDAPGSKYDKAVELGV 659  *M.leprae* SLAGFSRDDAKEAIVARGGKVAGSVSKKIAYVVVGDLPGYKYDKAVELGV 671  *M.lepromatosis* SLAGFSRDDAKEAIVARGGKVAGAVSKKIAYVVVGDLPGSKYDKAIELGV 663  *M.rhodesiae* SLPGFSRDEAKEAIITRGGKAVGSVSKKTSFVVAGDSPGSKYDKAVELGV 659  *M.parascrofulaceum* SLAGFSRDDAKEAIIARGGKAAGSVSKKTDYVVAGDAPGSKYDKAVKLGV 671  *M.smegmatis* SLAGFSRDQAKEAIIARGGKAAGSVSKKTAYVVAGDAPGSKYDKAVELGV 679 |

**References:**

1. Warbrick E, Heatherington W, Lane DP and Glover DM (1998) PCNA binding proteins in Drosophila melanogaster:the analysis of a conserved PCNA binding domain .

*Nucleic Acids Res* **26**: 3925–3932

1. Xu H, Zhang P, Liu L and Lee MYWT (2001) A novel PCNA-binding motif identified by the panning of a random peptide display library.

*Biochemistry* **40**: 4512-4520.

1. Dalrymple BP, Kongsuwan K, Wijffels G, Dixon NE and Jennings P A (2001) A universal protein–protein interaction motif in the eubacterial DNA replication and repair systems.

*Proc Natl Acad Sci USA* **98**: 11627–11632

1. Dalrymple BP, Wijffels G, Kongsuwan K and Jennings P (2003) Towards an understanding of protein-protein interaction network hierarchies. Analysis of DnaN (β)-binding peptide motifs in members of protein families interacting with the eubacterial processivity clamp, the β subunit of DNA Polymerase III. Conferences in Research and Practice in Information Technology **19**: Yi-Ping Phoebe Chen, Ed.
2. Haracska L, Acharya N, Unk I, Johnson RE, Hurwitz J, Prakash L and Prakash S (2005) A Single Domain in Human DNA Polymerase ι Mediates Interaction with PCNA: Implications for Translesion DNA Synthesis.

*Mol. Cell. Biol* **25**:1183-1190.
