# Supplementary material for: M. tuberculosis Sliding β-Clamp Does Not Interact Directly with the NAD+ -Dependent DNA Ligase
Source: PLoS One. 2012 Apr 24;7(4):e35702. doi: 10.1371/journal.pone.0035702 (PMC3335792; doi:10.1371/journal.pone.0035702)
Supplement: Figure S3 — 2Fo-Fc electron density map (blue mesh) contoured at 1σ around (A) Residues from 256–263 of Chain A and (B) Residues 184–186 (residues of the hydrophobic peptide binding groove). (DOC) [file pone.0035702.s003.doc]

Vandana Kukshal *et al.*, 2012

**Figure S3.**

2Fo-Fc electron density map (blue mesh) contoured at 1σ around (**A)** Residues from 256-263 of Chain A and **(B)** Residues 184-186 (residues of the hydrophobic peptide binding groove).

**(A)**

**(B)**
